# Target volume delineation and margins in the management of lung cancers in the era of image guided radiation therapy

**DOI:** 10.1002/jmrs.43

**Published:** 2014-02-24

**Authors:** Robert Lin

**Affiliations:** Innovative Informatics Pty LtdRevesby Heights, New South Wales, Australia

The goal of radiation therapy is to deliver a lethal dose of radiation to diseased tissue while minimising dose to surrounding healthy structures. Prior to the actual treatment delivery, treatment planning is the most critical part of a patient's radiation treatment management. The most crucial step is the accurate localisation and delineation of the target volume. Advances in tumour localisation and treatment delivery capabilities are limited by the inability to deliver treatment with complete precision to the localised tumour on a day-to-day basis over an entire course of radiation treatment. Most solid tumours are soft tissue masses, so the lack of inherent soft tissue contrast within images of intrathoracic regions can result in reduced visualisation and distinction of tumour boundaries from surrounding structures such as blood vessels, fatty tissues and lymph nodes.^1^ The ability to deliver an intended radiation dose to the tumour and to minimise the radiation dose to the healthy surrounding structures is related to the accuracy of the set up, tumour delineation, treatment plan generation and treatment delivery.^2^,^3^ These uncertainties significantly impact on the ability to deliver the intended dose to the target volume while minimising the dose to the surrounding structures. Due to these uncertainties, margins need to be added to the target volume as a buffer to accommodate the variation and uncertainties, and to ensure that the localised tumour receives the full intended dose. One of the most challenging aspects is the definition and delineation of the target volume.

There are many sources of uncertainty in radiation therapy which impact on treatment accuracy and patient outcome. Geometric uncertainties cause deviations between the intended dose and the actual dose received by the tumour volume. These uncertainties consist of both external and internal factors. The external factors relate to the external patient set up displacements and the internal influences are due to organ motion and respiration.^4^ The external set up displacement can be reduced with the application of immobilisation devices to fix the patient in the treatment position and by employing image guided radiation therapy (IGRT).^5^

The implementation of IGRT in radiation treatment has reduced the impact of organ motions and set up errors. Can the increased precision mitigate the issues associated with target volume delineation? I do not think so because IGRT is only as precise as the accuracy of the delineated target volume. The precision of IGRT and the steep dose gradient of the intensity modulated radiation therapy (IMRT) technique made accurate target volume delineation ever more important. The recent article by Liang et al. investigating the effect of IGRT on the margin between the clinical target volume (CTV) and planning target volume (PTV) in lung cancer found that the application of IGRT reduced the geometric uncertainties, but was unable to completely mitigate the errors.^6^ Proper identification and precise delineation of target volume improves accuracy whereas IGRT only improves precision.^7^

Current target volume delineation protocol is based on the guideline of International Commission on Radiation Units and Measurement (ICRU) recommendations for consistent definition of target volumes, but individualised based on tumour location, size, proximity to dose-limiting structure and the probability of high-grade treatment-related toxicities occurrence.^8^ The largest target volume variance tends to occur in the delineation of CTV because it includes the visible gross tumour as well as subclinical and microscopic invasions which are currently below the resolution limits of anatomical imaging techniques. This problem is exacerbated by adding margins based on assumptions derived from clinical experiences based on the known pathological pattern of spread and has high degree of uncertainty. Ketting et al. reported on a study assessing the consistency of the delineation of 3D target volumes between physicians and institutions on five lung adenocarcinomas and four nasopharyngeal squamous carcinomas cases. The study found, using observational qualitative analysis that the physicians varied in the margin placed around the CTV and the handling of concavities in the CTV. Furthermore, in analysing this data quantitatively, these variations resulted in statistically significant differences in the measured volumes of the physicians' PTVs.^9^

Target volume delineation and margin determination is even more challenging in lung when compared to other anatomical regions. The issue with overlapping anatomical structures that appears with similar densities on imaging scans making it difficult to distinguish, making target volume delineation highly imprecise and margin with high degree of variation. Liang et al. found the largest margin values were attributed to individual radiation oncologist's (RO) ability to identify and delineate the nodal invasion.^6^ A study conducted by Lin also found over and under estimation of mediastinal nodal margin was the most prominent problem causing moderate and major deviations.^10^

Uncertainties can be minimised by having concise contouring procedures and protocols, use of multimodality imaging techniques, training and multidisciplinary consultations either within the department or between institutions. A study by Senan et al. demonstrated the existence of statistically significant inter-observer variability with standardised contouring protocols and patients. The ratio of greatest delineated target volume over the smallest delineated target volume for gross tumour volume (GTV) of a T1N0 lung tumour was 1.6. Similarly, the ratio for PTV of a T2N2 lung tumour lesion was 2.^11^ However, despite every effort to minimise uncertainties and errors associated with target volume localisation and delineation, significant RO judgement is required in the interpretation of the computed tomography (CT) scans. Caldwell et al. found that significant inter-observer variability remained among experienced ROs, as different ROs will make different judgements and their judgements may vary from day to day. The published literature demonstrates that considerable inter-observer variation still exists, although with decreased magnitude, even with implemented standardised contouring protocols and the utilisation of multimodality imaging techniques.

Errors in target volume localisation and delineation occur early in the planning process and only once. This systematic error can produce the biggest deviation in the entire radiation therapy process and potentially can alter the outcome of the treatment. Once defined, this error is constant throughout the entire radiotherapy planning process and cannot be corrected unless a new target volume is defined and delineated and the patient treatment is re-planned.^12^

Based on the evidence presented, the question should be asked whether it is prudent to reduce the tumour volume margins.

To be able to answer that question, we must first examine the factors attributing to target volume delineation variability. The ability to accurately localise and delineate the target volume is based on the availability and the quality of imaging data and the clinical experience and expertise of the RO. With the advancement of imaging technology and technique, the visibility of tumour has increased which in turn increases the ability for ROs to delineate the borders of the malignancy as stated by Weiss and Hess. “*The contouring of a target volume is influenced to a largest extent by the observer's subjective interpretation of what he or she sees on the images*”.^13^ The utilisation of imaging modalities, in particular contrast enhanced CT, combined with positron emission tomography (PET) improve the ability of RO to identify a tumour and lymphatic nodal invasion from the surrounding structures. This results in the RO being able to define a more accurate and conformal target volume. Caldwell et al. found a reduction in target volume delineation variability when CT images were co-registered with PET for lung patients.

As far as the personal attribute is concerned, the training received, years of experience and the availability of instructions on contouring all have significant impact on tumour delineation especially of the extent of microscopic involvement. The implementation of multiple imaging modalities in the management of cancer has improved the visualisation of the tumour, but this has also created further problems for ROs as many have limited expertise, in particular the interpretation of PET images. The American Society for Therapeutic Radiology and Oncology (ASTRO) has recognised the problem by developing standardised delineating protocols for common cancers. Another professional institution recommended the development of close links between radiologists, nuclear medicine physicians and ROs to optimise the interpretation of radiological images to reduce the delineating variability and improve accuracy.^14^

Although target volume delineation can be significantly variable between different observers, I believe we are heading in the right direction in utilising the technology to be less subjective and less observer dependent in target volume delineation. This combined with precision in the treatment delivery and increased understanding of morphology and molecular profile of the tumour growth and pattern of spread has further improved the accuracy of target volume delineation leading to reduction in margins. Also with increased education and further training, the development of the standardised protocols and multidisciplinary collaboration has further reduced the degree of variability in target volume delineation.
